# *In vivo* comet assay in rabbit corneal epithelial cells following ocular instillation with genotoxic compounds

**DOI:** 10.1186/s41021-021-00184-4

**Published:** 2021-04-07

**Authors:** Haruna Tahara, Yoshinori Yamagiwa, Yu Haranosono, Masaaki Kurata

**Affiliations:** Research & Development Division, Senju Pharmaceutical Co., Ltd, 6-4-3, Minatojima-Minamimachi, Chuo-Ku, Kobe, Hyogo 650-0047 Japan

**Keywords:** Comet assay, Rabbit, Corneal epithelial cells, Genotoxicity test, Ophthalmic drug, Drug development

## Abstract

**Background:**

The *in vivo* comet assay is used to evaluate the genotoxic potential of compounds by detecting DNA strand breaks in cells isolated from animal tissue. The comet assay of hepatocytes is well established; however, the levels of systemic drug exposure following systemic administration are often insufficient to evaluate the genotoxic potential of compounds on the ocular surface following ocular instillation. To investigate the possibility of using the comet assay as a genotoxic evaluation tool for the ocular surface, we performed this assay on the corneal epithelial cells of rabbit eyes 2 h after the single ocular instillation of five genotoxic compounds, namely ethidium bromide, 1,1′-dimethyl-4,4′-bipyridinium dichloride (paraquat), methyl methanesulfonate (MMS), acrylamide, and 4-nitroquinoline 1-oxide (4-NQO).

**Results:**

The mean % tail DNA, as an indicator of DNA damage, in the corneal epithelial cells treated with ethidium bromide, MMS, and 4-NQO exhibited statistically significant increases compared with those in the negative controls (saline or 5 % dimethyl sulfoxide in saline). However, paraquat and acrylamide did not increase the mean % tail DNA, presumably because of the high antioxidant levels and low cytochrome P450 levels present in the corneal epithelium, respectively.

**Conclusions:**

The comet assay was able to detect genotoxic potential on the ocular surface following ocular instillation with genotoxic compounds. The study findings indicate that the *in vivo* comet assay may provide a useful tool for assessing the genotoxicity of compounds topically administrated on the ocular surface under mimicking clinical condition.

## Introduction

The eye is an important sensory organ, especially for maintaining quality of life, given that humans obtain approximately 80 % of external information from their vision [1]. Eye-drop drugs are widely used for diagnosis, the treatment of eye diseases, and the alleviation of eye discomfort. During ophthalmic drug development, nonclinical toxicity tests of ophthalmic formulations are generally conducted to clarify the toxicity profile of the constituent compounds, mainly with respect to their target organ/tissue, and to determine the relationship between toxicity and the dose or systemic exposure levels. The genotoxicity test is a particularly important toxicity test because compound genotoxicity can result in carcinogenesis. The International Conference on Harmonisation (ICH) guidance on genotoxicity testing and data interpretation for pharmaceuticals intended for human use describes two options for the standard battery of genotoxicity testing [ICH S2(R1)] [2], and genotoxicity tests of either Option 1 or 2 must be performed. Option 1 includes two *in vitro* tests (a bacterial mutation test and a mammalian cell genotoxicity test) and one *in vivo* test, while Option 2 includes one *in vitro* test (a bacterial mutation test) and two *in vivo* tests. In addition, if an *in vitro* test using mammalian cells is positive in Option 1, additional *in vivo* tests are recommended. For the *in vivo* genotoxicity tests, a micronucleus test with bone marrow-derived erythrocytes [3] and a comet assay with hepatocytes [4, 5] are commonly performed. These genotoxicity tests mainly evaluate the genotoxic potential of a compound toward target tissues (e.g., bone marrow or liver) following systemic administration such as oral or intravenous administration. However, ophthalmic solutions directly expose the ocular surface to high concentrations of compounds. In this situation, the exposure levels of the ocular surface (e.g., cornea) following ocular instillation are often higher than those of other tissues (e.g., bone marrow and liver) following oral or intravenous administration. For this reason, the exposure levels of tissues such as the bone marrow and liver following systemic administration are insufficient to evaluate the genotoxic potential on the ocular surface following ocular instillation. Therefore, another genotoxicity test may be desirable to assess the genotoxic potential of compounds on the ocular surface.

To the best of our knowledge, there have been few reports to date regarding genotoxicity tests on the ocular surface after ocular instillation. Recently, we investigated the *in vivo* unscheduled DNA synthesis (UDS) test using rabbit corneas after ocular instillation (*in vivo* corneal UDS test) [6]. However, a radioisotope analysis facility is required to perform the UDS test. To investigate the application of a commonly used method that does not rely on radioisotopes, we focused on the comet assay for ocular genotoxicity testing.

A comet assay detects single- or double-strand DNA breaks and alkali-labile sites through the electrophoresis of single-cell suspensions under alkaline conditions [7, 8]. The quantity of DNA that migrates by electrophoresis indicates the level of DNA damage in the individual cell. A guideline for the *in vivo* mammalian alkaline comet assay was issued in 2016 by the Organisation for Economic Co-operation and Development (OECD) [9], and this test has been widely adopted since then. Moreover, the *in vitro* comet assay using primary corneal epithelial cells has already been reported [10]; therefore, the appropriate technique for isolating corneal epithelial cells from the eye is known. Thus, the comet assay may be applicable for the evaluation of *in vivo* genotoxicity on the ocular surface. Regarding the animal species used, we selected rabbits for this study, because rabbits are commonly used in ocular toxicity studies in drug developments [11, 12] and have also been used for the *in vivo* corneal UDS test [6].

In this study, we used five well-known genotoxic compounds with different mechanisms of genotoxicity: ethidium bromide as a DNA intercalator [13]; 1,1′-dimethyl-4,4′-bipyridinium dichloride (paraquat) as a radical generator [14]; methyl methanesulfonate (MMS) as an alkylating agent [15]; and acrylamide and 4-nitroquinoline 1-oxide (4-NQO) as bulky DNA adduct-forming agents [16, 17]. Except for MMS, all of these compounds were used in the *in vivo* corneal UDS test [6], and were selected for comparisons with the results of the previous test. MMS was used in the present study because it is often used as a positive control agent in the *in vivo* mammalian alkaline comet assay [9].

## Materials and methods

### Chemicals

Ethidium bromide (CAS No. 1239-45-8), paraquat (CAS No. 1910-42-5), MMS (CAS No. 66-27-3), acrylamide (CAS No. 79-06-1), and 4-NQO (CAS No. 56-57-5) were used as the test compounds. These compounds were purchased from FUJIFILM Wako Pure Chemical Corporation (Osaka, Japan). Saline and dimethyl sulfoxide (DMSO) were obtained from Otsuka Pharmaceutical Factory (Tokushima, Japan) and Nacalai Tesque, Inc. (Kyoto, Japan), respectively. Saline or saline containing 5 % DMSO (5 % DMSO) were used as the negative controls.

### Animals and husbandry

Male Japanese white rabbits (Kbs:JW) were purchased at 10–11 weeks of age from Kitayama Labes, Co., Ltd. (Nagano, Japan), and the test compounds were administered at 11–12 weeks of age (bodyweight 2.0–2.4 kg). The rabbits were individually housed in air-conditioned rooms with a temperature between 19 and 25 °C, relative humidity of 40–70 %, and a 12-h light/dark cycle. Each rabbit was provided with commercial pellet feed (Labo R Stock, Nosan Corporation, Kanagawa, Japan) and supplied with tap water *ad libitum*. A dumbbell made from polypropylene (Bio-Serv, Flemington, NJ) was placed in each cage as an environmental enrichment device. The animals were acclimated for at least 5 days before the experiments. All experimental procedures were in accordance with the guidelines for animal experimentation at Senju Pharmaceutical Co., Ltd., and the protocol was reviewed by the Institutional Animal Care and Use Committee (IACUC) of Senju Pharmaceutical Co., Ltd.

### Procedures for animal treatments

Thirty-four clinically and ophthalmologically normal rabbits were randomly assigned to each treatment group (2–4 animals/group). The experiments were performed as 3 phases (Tests 1–3). To prepare the dosing solutions, ethidium bromide, paraquat, MMS, and acrylamide were dissolved in saline, while 4-NQO was prepared in 5 % DMSO. Based on the results of the preliminary eye irritation tests and the *in vivo* corneal UDS test [6], the concentration without abnormal irritative or histopathological changes was selected as the high dose for each compound, and then the low dose was set with a common ratio of 4 or 5.

The 0.25 and 1 % ethidium bromide, 1 and 5 % paraquat (Test 1), 0.6 and 3 % MMS, 0.6 and 3 % acrylamide (Test 2), and 0.2 and 1 % 4-NQO (Test 3) were instilled once onto the eyes of each rabbit at a dosing volume of 50 µL per eye. For the negative controls, saline (Tests 1 and 2) and 5 % DMSO (Test 3) were administered in the same manner. For the groups treated with 1 % ethidium bromide, 0.2 and 1 % 4-NQO, or the 5 % DMSO negative control, these dosing solutions were administered to only the right eyes of four rabbits (i.e., four eyes per group), because slight eye irritation was observed with these dosing solutions during gross observation of the preliminary eye irritation tests. The 0.25 % ethidium bromide and other dosing solutions were administered to both eyes of two rabbits (four eyes per group). After ocular instillation, the eyelids were artificially blinking several times. The rabbits were euthanized 2 h after ocular instillation with an overdose (approximately 90 mg/kg) of intravenously injected thiopental solution (Ravonal; Nipro ES Pharma Co. Ltd., Osaka, Japan). After euthanasia, the eyeballs were collected from the rabbits. The timing of collection was set to the same as that used in the previous *in vivo* corneal UDS test [6]. For each treatment group, three eyes were subjected to the comet assay and the remaining one eye was subjected to histopathological examination.

### Isolation of corneal epithelial cells for the comet assay

The collected eyes (three eyes per group) were washed with Ca^2+^- and Mg^2+^-free phosphate-buffered saline [PBS(−)]. After washing, the corneas were removed from the eyes with a scissor and tweezer. The removed corneas were treated with 1.2 unit/mL Dispase II (Roche Diagnostics GmbH, Mannheim, Germany) in Minimum Essential Media (MEM) (Thermo Fisher Scientific K. K., Tokyo, Japan) supplemented with 10 % bovine serum (10 % BS/MEM) overnight at 4 °C. The corneal epithelial cells were isolated from the corneas with a spatula and placed into fresh 10 % BS/MEM. The cells were centrifuged at 140 ×*g* for 5 min and the supernatants were discarded. The cells were resuspended with 1 mL of 0.25 % trypsin (Thermo Fisher Scientific K. K.) and incubated for 10 min at 37 °C. A further 8 mL of 10 % BS/MEM was added to the cells, and the cell suspensions were passed through a 70-µm cell strainer. The cells were centrifuged at 140 ×*g* for 5 min and the supernatants were discarded. The cells were then resuspended with PBS(−) to a density of approximately 2 × 10^5^ cells/mL.

### Alkaline comet assay

The alkaline comet assay was conducted according to a previously published method [18]. A 30 µL of prepared cell suspension was mixed with 270 µL of melted agarose solution (CometAssay LMAgarose; Trevigen, Inc., Gaithersburg, MD). Then, 30 µL of this mixture was placed on each well of a 20-well slide (Comet Slide HT, Trevigen, Inc.), and the slides were left for approximately 10 min at 4 °C to harden the agarose. Two slides per eye were prepared. The slides were immersed in lysis solution containing 2.5 M sodium chloride, 100 mM ethylenediaminetetraacetic acid (EDTA), 10 mM tris(hydroxymethyl)aminomethane hydrochloride (Tris HCl), and 1 % (v/v) polyethylene glycol mono-*p*-isooctylphenyl ether for 1 h at 4 °C. The slides were subsequently immersed in the alkaline unwinding solution (200 mM sodium hydroxide and 1 mM EDTA, pH > 13) for 20 min at room temperature. Electrophoresis was performed with the same solution at 1 V/cm for 30 min under refrigeration. After electrophoresis, the slides were washed twice with ultrapure water, and dehydrated by immersion in ethanol for 10 min. The slides were stained with SYBR Green I Nucleic Acid Gel Stain (excitation maxima at 497 nm, emission maxima at 520 nm; Thermo Fisher Scientific K. K.) diluted 1:1,000 with Tris-EDTA buffer (pH 7.5), and then mounted using ProLong Gold (Thermo Fisher Scientific K. K.). The slides were observed using a BX51 fluorescence microscope (Olympus Corporation, Tokyo, Japan) with the NIBA filter (excitation at 460–495 nm and emission at 510–550 nm) equipped with a CCD camera (scA1300-32 fm; Basler AG, Ahrensburg, Germany).

First, the number of “hedgehogs” was counted among 100 comets per eye (300 comets per group). According to the Atlas of Comet Assay Images, hedgehogs are highly fragmented cells that present as a small or non-existent comet head and large diffuse comet tail under microscopy [19]. Second, 100 scorable comets (i.e., with a clearly defined head and tail with no interference from neighboring cells) without hedgehogs were measured per eye (300 comets per group). The percentage of tail DNA (% tail DNA) (DNA fluorescence intensity in the tail/total DNA fluorescence intensity × 100) was measured as an indicator of DNA damage using the Comet Assay IV software, version 4.3.2 (Perceptive Instruments, Haverhill, UK).

### Microscopic examination of the corneal tissue

The collected eyes (one eye per group) were fixed with 1 % formaldehyde/2.5 % glutaraldehyde in 0.1 M phosphate buffer fixative overnight at 4 °C, and then post-fixed with 10 % neutral-buffered formalin solution. The tissues were dehydrated using a graded alcohol series and embedded in paraffin. Approximately 3-µm-thick corneal tissue sections were prepared and stained with hematoxylin and eosin. All microscopic images were obtained with a BX51 microscope fitted with a DP74 digital camera (Olympus Corporation), and the images were analyzed using cellSens Standard imaging software, version 2.3 (Olympus Corporation).

### Statistical analysis

The mean and standard deviation (SD) of the % tail DNA were calculated for all experimental groups. The data were assumed to have a normal distribution and homogeneous variance. Dunnett’s multiple comparisons test (one-tailed) was used to compare the mean value of each of the test compound groups with that of the negative control group (saline for Tests 1 and 2, and 5 % DMSO for Test 3). JMP version 13.2.1 (SAS Institute Japan, Ltd., Tokyo, Japan) was used for all statistical analyses. Probability (*p*) values of < 0.05 were considered statistically significant.

## Results

### Comet assay of corneal epithelial cells

**Fig. 1:**
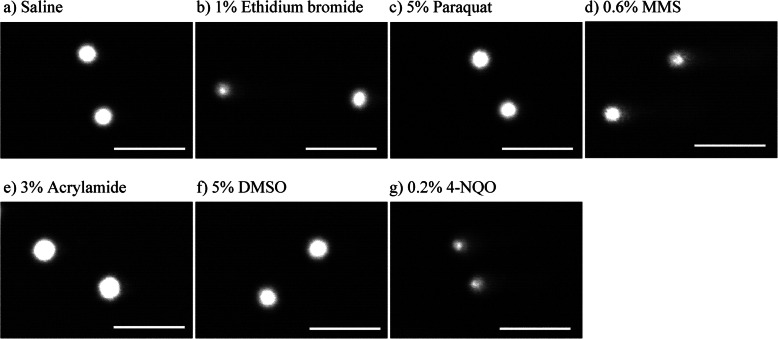
Representative comet images of corneal epithelial cells in rabbits. The comet assay were performed using corneal epithelial cells 2 h after the single ocular instillation of saline as the negative control (**a**), 1 % ethidium bromide (**b**), 5 % 1,1′-dimethyl-4,4′-bipyridinium dichloride (paraquat) (**c**), 0.6 % methyl methanesulfonate (MMS) (**d**), 3 % acrylamide (**e**), 5 % dimethyl sulfoxide in saline (5 % DMSO) as the negative control (**f**), or 0.2 % 4-nitroquinoline 1-oxide (4-NQO) (**g**). The corneal epithelial cells were stained with SYBR Green I Nucleic Acid Gel Stain. Scale bars: 100 μm

**Fig. 2:**
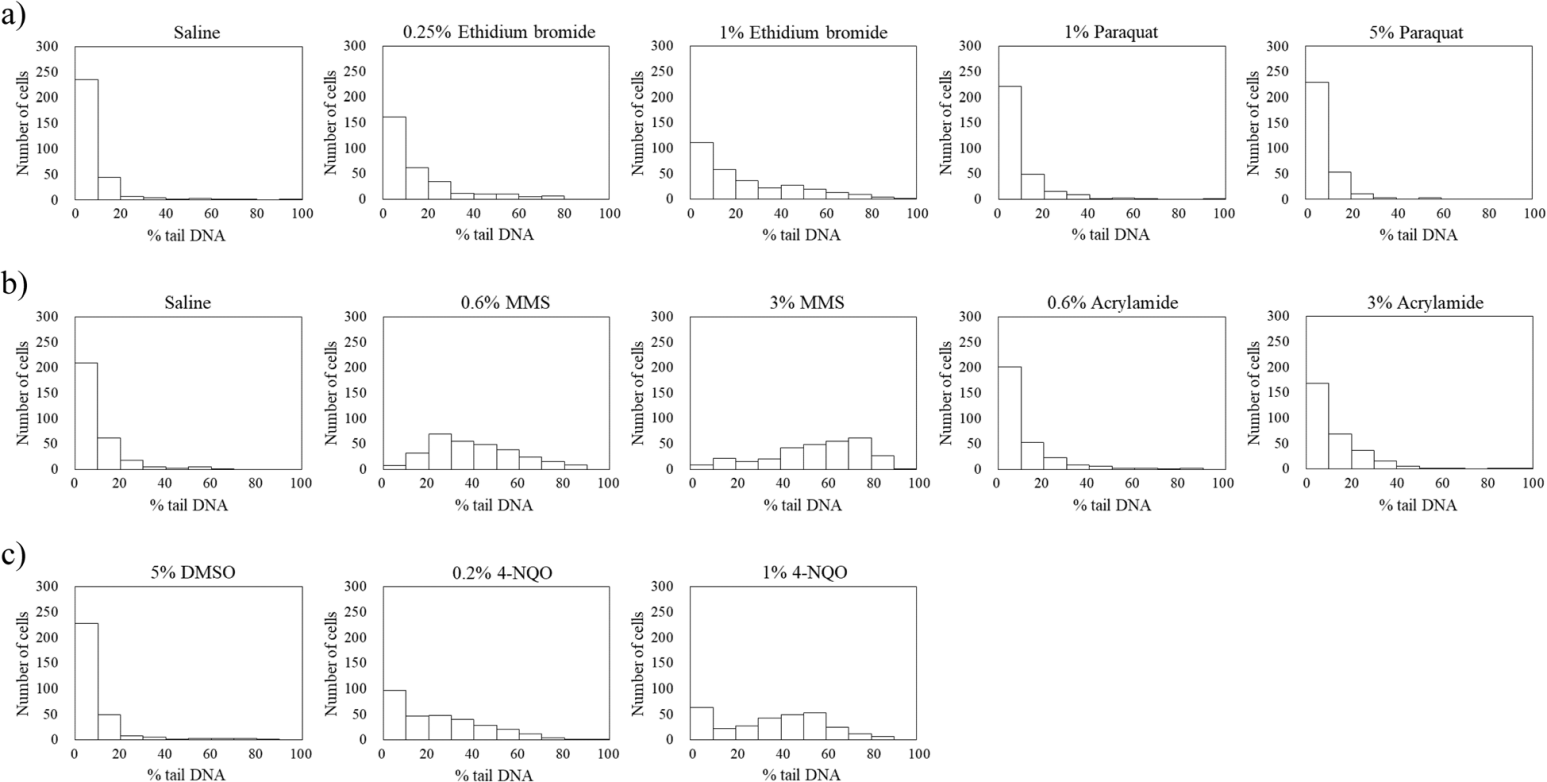
Distribution of the % tail DNA of the total number in corneal epithelial cells (*n *= 3). The eyes were treated with an ocular instillation of ethidium bromide, 1,1′-dimethyl-4,4′-bipyridinium dichloride (paraquat) in the Test 1 (**a**), methyl methanesulfonate (MMS), acrylamide in the Test 2 (**b**), or 4-nitroquinoline 1-oxide (4-NQO) in the Test 3 (**c**). For the negative controls, saline (**a**, **b**) and 5 % DMSO (**c**) were administered in the same manner

Figure 1 shows representative comet images of the rabbit corneal epithelial cells treated with ethidium bromide, paraquat, MMS, acrylamide, 4-NQO, and the negative controls (saline and 5 % DMSO). Figure 2 shows the distribution of the values of % tail DNA in corneal epithelial cells in pooled data from three eyes. In the saline- and 5 % DMSO-treated corneal epithelial cells as the negative groups, most cells showed the values of % tail DNA within a range of 0–20 %. In the ethidium bromide, MMS, and 4-NQO treated groups, the number of cells with the high values of % tail DNA increased compared to the negative control-treated cells. In the paraquat- and acrylamide-treated groups, the values of % tail DNA show similar distribution to the negative control. The mean % tail DNA and the frequency of hedgehogs for each of these compounds and controls are shown in Table 1. Statistically significant increases in the mean % tail DNA were observed following treatment with ethidium bromide, MMS, and 4-NQO compared with the negative controls. These increases generally appeared to be dose-dependent. No significant increases in % tail DNA were observed after ocular instillation with paraquat or acrylamide. In the 1 % ethidium bromide-, 3 % MMS-, and 1 % 4NQO-treated eyes, the numbers of hedgehog-shaped corneal epithelial cells were slightly increased compared with those in the negative controls. Few hedgehogs were observed in the other samples.

**Table 1:** Mean % tail DNA and hedgehog frequency in the corneal epithelial cells

Compounds	Concentration	Number of eyes	% tail DNA (Mean ± SD)			Hedgehog (%) (Mean ± SD)		
Test 1								
Saline	0 %	3	7.3	±	0.4	0.0	±	0.0
Ethidium bromide	0.25 %	3	15.2	±	1.9^a^	0.7	±	1.2
	1 %	3	24.0	±	1.6^a^	7.0	±	5.6
Paraquat	1 %	3	8.0	±	2.5	0.0	±	0.0
	5 %	3	6.8	±	1.2	0.0	±	0.0
Test 2								
Saline	0 %	3	8.8	±	2.2	0.0	±	0.0
MMS	0.6 %	3	39.9	±	6.0^a^	0.0	±	0.0
	3 %	3	55.1	±	3.9^a^	7.3	±	4.6
Acrylamide	0.6 %	3	10.3	±	5.2	0.0	±	0.0
	3 %	3	12.1	±	1.7	0.0	±	0.0
Test 3								
5 % DMSO	0 %	3	7.9	±	2.0	0.0	±	0.0
4-NQO	0.2 %	3	24.6	±	3.2^a^	1.3	±	1.2
	1 %	3	36.0	±	11.6^a^	6.7	±	4.2

^a^: Significantly higher than the negative control at the probability of 5 % level (Dunnett’s multiple comparison test, one-tailed)

Paraquat: 1,1′-dimethyl-4,4′-bipyridinium dichloride, MMS: methyl methanesulfonate, 5 % DMSO: 5 % dimethyl sulfoxide in saline, 4-NQO: 4-nitroquinoline 1-oxide

### Microscopic examination of rabbit corneal tissue

**Fig. 3:**
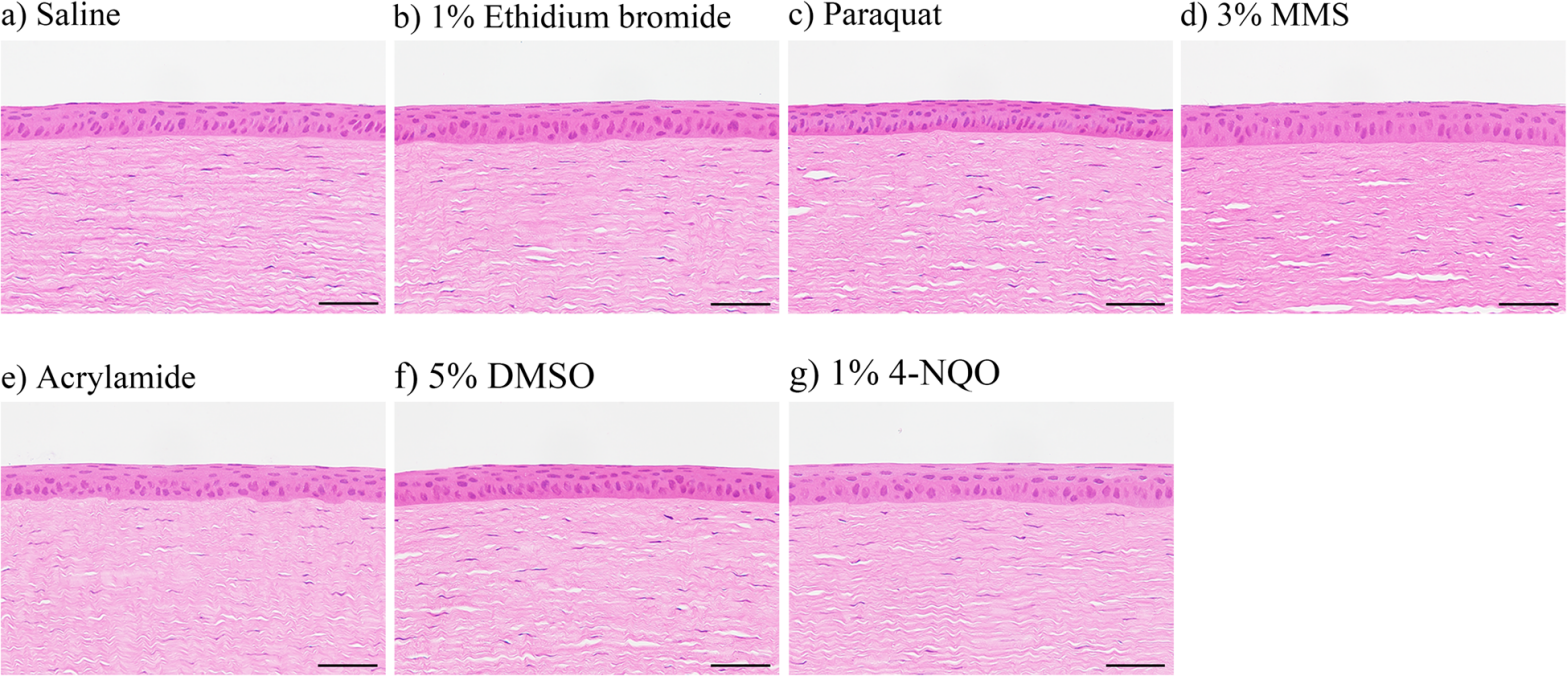
Representative photomicrographs of corneal epitheliums in rabbits. The animals were treated with saline as the negative control (**a**), 1 % ethidium bromide **(b**), 5 % 1,1′-dimethyl-4,4′-bipyridinium dichloride (paraquat) (**c**), 3 % methyl methanesulfonate (MMS) (**d**), 3 % acrylamide (**e**), 5 % dimethyl sulfoxide in saline (5 % DMSO) as the negative control (**f**), or 1 % 4-nitroquinoline 1-oxide (4-NQO) (**g**). The corneas were collected 2 h after dosing, and corneal sections were stained with hematoxylin and eosin. No histopathological changes were observed for any of the treatment or control groups. Scale bars: 50 μm

Figure 3 depicts representative photomicrographs of hematoxylin and eosin staining of corneal epithelium sections following treatment with the test compounds and controls. No histopathological change in the corneal epithelium was observed for any of the treatment or control groups.

## Discussion

In this study, we performed comet assay on the corneal epithelial cells of rabbit eyes following the single ocular instillation of five genotoxic compounds. The means % tail DNA were 7.3 and 8.8 % in the saline treated eyes as the negative controls in Tests 1 and 2, respectively. In the 5 % DMSO treated eyes, the mean % tail DNA was 7.9 % in Test 3, and the value was similar to those of the saline treated groups. In addition, distribution of the values % tail DNA of corneal epithelial cells treated with negative controls was comparable among these groups. In the JaCVAM international validation trial of the *in vivo* comet assay, the % tail DNA in negative control groups set within the ranges of 1–8 % and 1–20 % in the liver and stomach, respectively [20]. In the *in vivo* comet assay using the mouse skin, the means % tail DNA in negative control groups were 12.3–15.5 % [21]. Moreover, the means % tail DNA were 10–20 % in our previously reported an *in vitro* comet assay using a three-dimensional (3D) corneal model [18]. Because the values of % tail DNA in the present study were close to the ranges of negative control values in these studies, we judged that this study was appropriately performed.

Statistically significant increases in the % tail DNA in the corneal epithelial cells were observed in ethidium bromide-, MMS-, and 4-NQO-treated eyes compared with those of the negative controls. The results for these compounds were consistent with our previously reported *in vivo* corneal UDS test [6] and an *in vitro* comet assay using a 3D corneal model under conditions mimicking ocular instillation [18]. However, the numbers of hedgehogs were slightly increased (approximately 7 %) after treatments with 1 % ethidium bromide, 3 % MMS, and 1 % 4-NQO in the present study. It has been reported that hedgehogs may represent the early stages of apoptosis during which DNA damage may yet be repairable, therefore should not be taken as an indication of cytotoxicity [22]. Furthermore, the OECD guideline for conducting *in vivo* comet assay proposes that organ/tissue histopathological changes be used as an evaluation item of cytotoxicity [9]. In this study, no histopathological change was observed in the corneal epithelium for any test compound. For these reasons, the increased % tail DNA for groups treated with ethidium bromide, MMS, and 4-NQO in this study were most likely caused by DNA damage instead of cytotoxicity. In addition, since distribution of the values of % tail DNA in corneal epithelial cells was within the range of 0–20 % at some cells in the ethidium bromide- and 4-NQO-treated groups, it suggests that there were some normal corneal epithelial cells with slight DNA damage. In contrast, since few cells had distribution of the values of % tail DNA within the range of 0–20 % in the MMS-treated group, suggesting that most cells caused DNA damage.

Acrylamide did not induce DNA damage in this study in agreement with results previously reported for the *in vivo* corneal UDS test [6] and the *in vitro* comet assay using the 3D corneal model [18]. Acrylamide is known to show genotoxicity in the *in vivo* comet assay of hepatocytes after oral administration in rats [23]. Acrylamide is known to be metabolized by cytochrome P450 (CYP) 2E1, and its metabolites have been shown to induce DNA damage through the formation of bulky DNA adducts [16]. However, the mRNA expression of CYP in the cornea is considerably lower than that in the liver [24], suggesting that the generation of genotoxic metabolites is low on the ocular surface.

No increases in DNA migration during electrophoresis were observed in the 5 % paraquat-treated eyes in this study. Paraquat is reduced by nicotinamide adenine dinucleotide phosphate (NADPH)-cytochrome P450 reductase, generating free radicals that include reactive oxygen species and leading to cytotoxicity and DNA damage [14, 25]. The corneal epithelial cells contain abundant antioxidants such as superoxide dismutases, catalase, and glutathione peroxidases [26]. Therefore, the generation of radicals by paraquat might have been suppressed in these cells by antioxidants, thereby preventing DNA damage at the ocular surface.

Paraquat has been reported to cause formation of micronuclei in the bone marrow cells after intraperitoneal administration in mice [27]. In addition, paraquat shows a slight increase in DNA damage in the *in vivo* rabbit corneal UDS test under the same conditions of administration (i.e., instillation of 5 % paraquat) [6]. A possible factor of the cause of differences is change of ocular surface conditions affected by systemic anesthesia to the animals. Ocular instillation to the rabbit was performed without anesthesia in the present study, whereas rabbits were anesthetized before ocular instillation in the *in vivo* corneal UDS test. Generally, ophthalmic solutions following instillation are immediately diluted with tear fluid, and excreted from the ocular surface through nasolacrimal duct by tear turnover [28–30]; however, the turnover rate of the tear fluid under anesthesia is extremely low or essentially zero [31]. Thus, the exposure levels on the ocular surface following instillation under anesthesia may have been higher than those without anesthesia.

The guidance of ICH S2(R1) describes some cases, such as topically applied drugs with low systemically exposure, can be evaluated for genotoxicity on the applied site even though it has not yet been widely used [2]. Therefore, in case that the *in vitro* genotoxicity tests of compounds using mammalian cells show positive results in the ophthalmic drug development, the present test system could provide an additional *in vivo* testing tool using the ocular surface when the exposure level following systemic administration is not achieved the level on the ocular surface.

The eyes were collected from rabbits 2 h following ocular instillation. Because UDS was detected on the corneal epithelial cells 2 h after instillation in the *in vivo* corneal UDS test [6], it is considered that DNA damage and subsequent repair have been occurred within 2 h. Even though it is necessary further investigation to set the optimal timing, tissue sampling for analysing comet assay is considered to be performed before timing of DNA repair. In addition, since the present study aimed research/investigation work, it does not partially meet the OECD guideline for conducting *in vivo* comet assay [9]; for example, acquisition of historical data of the negative controls, the number of cells examined per eye, and the number of used animals per group. Further validations are required for these points in the future.

Attention should also be paid to the genotoxicity mechanism of the test compounds in performing the comet assay. The comet assay can detect the genotoxic potential of compounds that are categorized as DNA intercalators, alkylating agents, and bulky DNA adduct-forming agents, but not reliably detect genotoxic potential of DNA-crosslinking agents [32]. When a compound has a DNA crosslinking potential, the relative reduction of DNA migration induced by a strand-breaking agent should be indirectly measured by a modified comet assay [33–35]. For this reason, researchers need to choose the appropriate genotoxicity test with consideration of the mode of action of the compounds being tested.

## Conclusions

The comet assay detected DNA damage in the corneal epithelial cells of rabbit eyes after the ocular instillation of known genotoxic compounds. This method offers an additional *in vivo* genotoxicity test for the development of ophthalmic drugs under conditions that mimic clinical settings.

## Data Availability

All data generated or analyzed during this study are included in this published article. All materials used in this study were described exactly in the article.
